# Isolated Hilar Mass in Unicentric Castleman Disease: Surgery As the Definitive Diagnosis and Treatment

**DOI:** 10.7759/cureus.92649

**Published:** 2025-09-18

**Authors:** Saif Ahmad, Syed Mashood Iqbal, Rabab Qazi, Evangeline Buck, Awab Ismail, Nadeem Maddekar, Maninder Kalkat

**Affiliations:** 1 Maxillofacial Surgery, Royal Stoke University Hospital, University Hospitals of North Midlands NHS Trust, Stoke-on-Trent, GBR; 2 Acute Medicine, Queen Elizabeth Hospital Birmingham, Birmingham, GBR; 3 Respiratory Medicine, Queen Elizabeth Hospital Birmingham, Birmingham, GBR; 4 Endocrinology and Diabetes, Hereford County Hospital, Hereford, GBR; 5 Cardiothoracic Surgery, Queen Elizabeth Hospital Birmingham, Birmingham, GBR

**Keywords:** anterior mediastinal mass, bilobectomy, cardiothoracic surgery, diagnostic challenge in lymphoma, fdg pet ct, ga-68 dotatate scan, hilar mass, histopathology (hp), mediastinum malignancy, unicentric castleman disease

## Abstract

Castleman disease is a rare group of benign B-cell lymphoproliferative disorders characterised by enlarged lymph nodes, presenting either as unicentric, involving a single enlarged lymph node, or multicentric, involving multiple lymph nodes. Symptoms range from mild to life-threatening. Although it is considered a benign condition, it needs to be differentiated from similar presenting malignancies such as carcinoid tumour and lymphoma. This differentiation often presents a clinical challenge. We present a case of a 47-year-old male who presented with lower back pain and mild shortness of breath after a fall. On routine imaging, a 6.4-cm mass was incidentally found. Biopsies and both fluorodeoxyglucose positron emission tomography (FDG PET) and gallium-68 DOTA-[Tyr³]-octreotate (DOTATATE) positron emission tomography proved inconclusive in diagnosing the mass. In this case, diagnostic certainty was only gained after an en bloc bilobectomy. We discuss the challenges and options of gaining a definitive diagnosis, while considering the risks and benefits of invasive methods such as surgery in what is considered a benign disease. However, in this case, surgery resulted in complete resolution of symptoms and positive results at follow-up.

## Introduction

Castleman disease is an uncommon disorder characterised by non-neoplastic proliferation of lymphoid tissue. This definition is expressed throughout the literature, including in a similar case report by Gomez-Ramirez et al. in the *American Journal of Cancer Prevention* [1]. Diagnosis is often challenging, as the clinical course often mimics other diseases causing lymphadenopathy. The varying disease course and common differential diagnoses are explored by Ehsan and Zahra in a comprehensive review article in *StatPearls* [2].

It presents as two distinct subtypes: the more common unicentric (highlighted by Gomez-Ramirez et al. in a similar case report [1]) or localised Castleman disease (CD), involving a single lymph node and rarely any systemic features, or less common multicentric CD, characterised by multiple node involvements and systemic manifestations, which was explored in other case reports such as Linkhorn et al. and Carbone et al. in their respective case reports [3,4].

Studies found that there is no gender difference in unicentric Castleman disease (UCD), while men are slightly more likely to be afflicted with multicentric Cattleman disease (MCD). The demographic distribution of the condition, as well as the difference in prevalence of the subtypes, is explored in detail by Dispenzieri and Fajgenbaum in a comprehensive overview of the condition in an article in the *American Society of Hematology* [5]. As outlined, plasma-cell types (10%) and hyaline vascular (90%) are the two primary histological variants. Although the exact aetiology remains unknown, MCD is closely associated with HIV and human herpesvirus 8 (HHV-8), with the disease process being driven by the dysregulation of inflammatory cytokines, namely interleukin-6 (IL-6). The theorised pathophysiology is explored in detail by Ehsan et al. [2].

UCD can sometimes present atypically, such as with a retroperitoneal mass, as in a case report by Ashjaei et al. [6], in which a child presented with failure to thrive due to such a mass. A similar atypical presentation was reported by Muhammad et al. [7]. Clinically, UCD presents as a solitary mass, most commonly in the mediastinal region (70%), which remains asymptomatic and picked up on routine imaging, often carrying a good prognosis [5]. The common presentation of UCD as mediastinal involvement is seen in the literature, such as in the comprehensive overview of the condition by Ehsan et al. [2].

Diagnosis is based on the fulfilment of both major and minor criteria, including the presence of an enlarged lymph node and histological confirmation. The major and minor diagnostic criteria are explained by Dispenzieri and Fajgenbaum in their comprehensive overview article [5]. However, definitive diagnosis is based on post-operative pathological findings, and the importance of histopathology examination of tissue is iterated in multiple case reports, such as in the case report by Janoud et al. [8]. As expressed in their case report, the best available treatment for UCD remains surgical resection, while MCD often responds to Rituximab and other interleukin-6 (IL-6) targeted therapies [8]. Discussed below is a challenging case wherein multiple imaging modalities and pre-operative biopsies did not yield a definitive diagnosis due to the significant overlap with other pathologies such as lymphoma and carcinoid tumours.

## Case presentation

**Figure 1:**
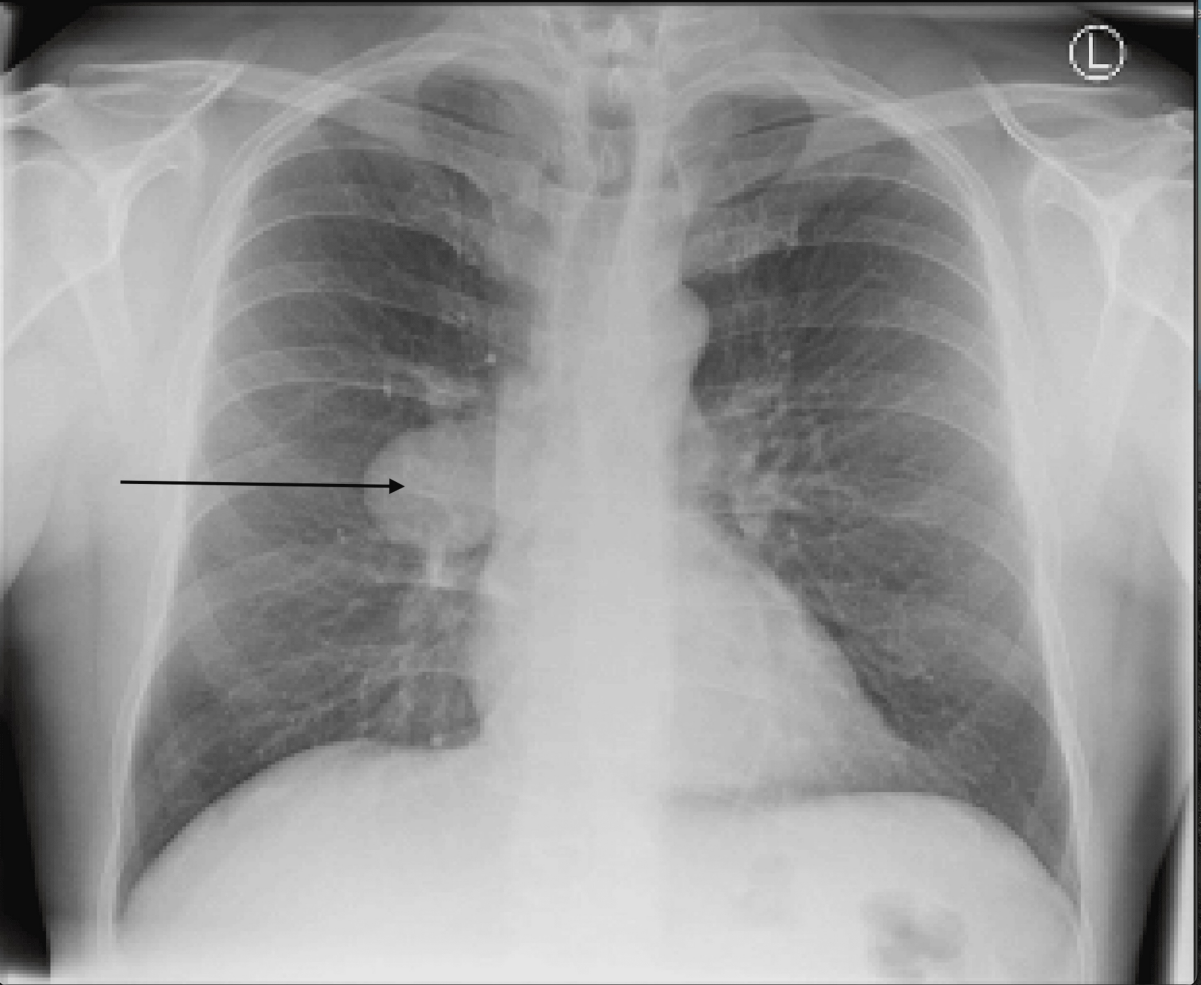
Chest radiograph in PA view, erect, showing a well-defined rounded density overlying the right hilum medially (black arrow). PA: posteroanterior view.

**Figure 2:**
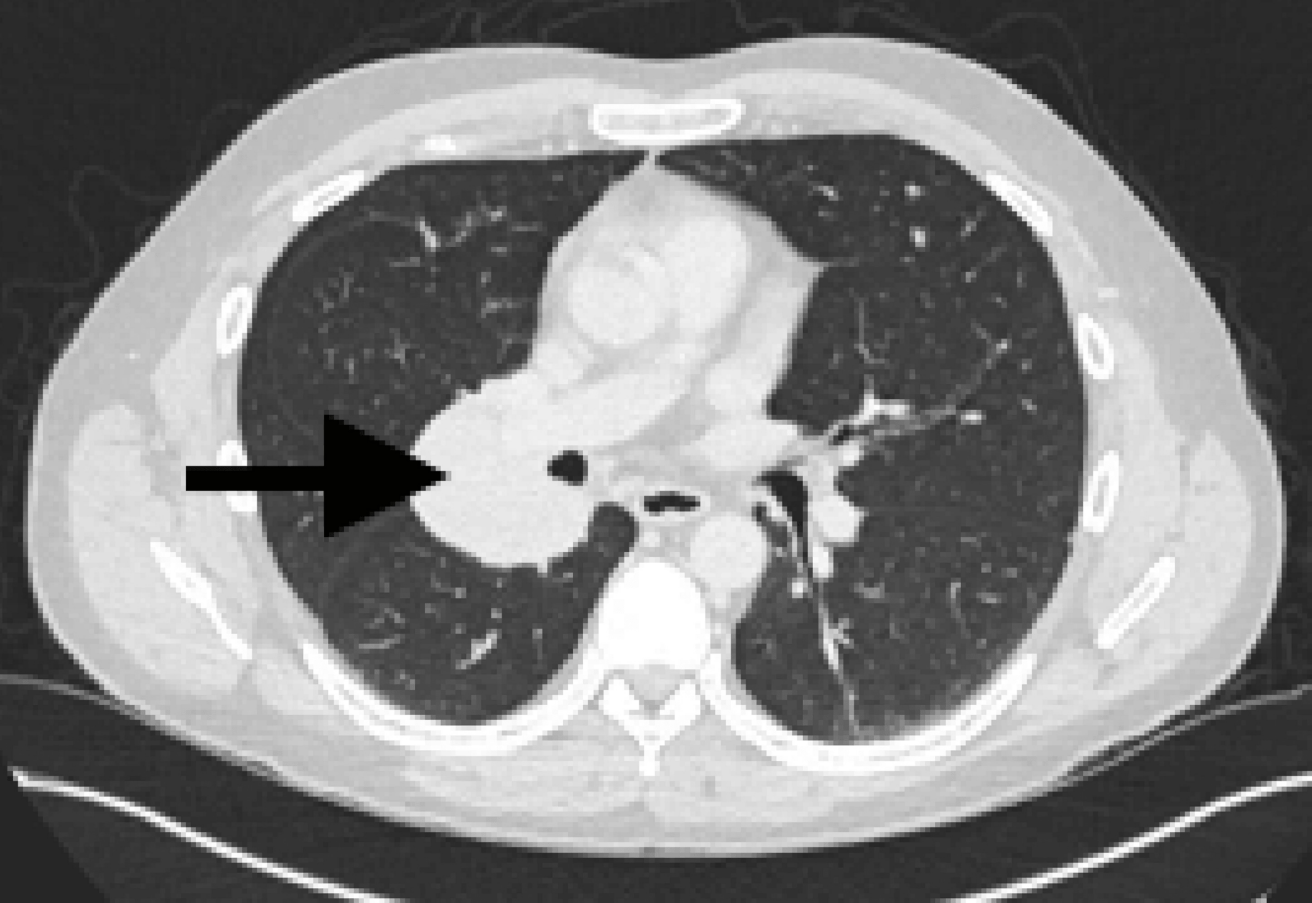
Computerised tomography (CT) in axial view showing a well-defined 6.4-cm right hilar mass (black arrow).

**Figure 3:**
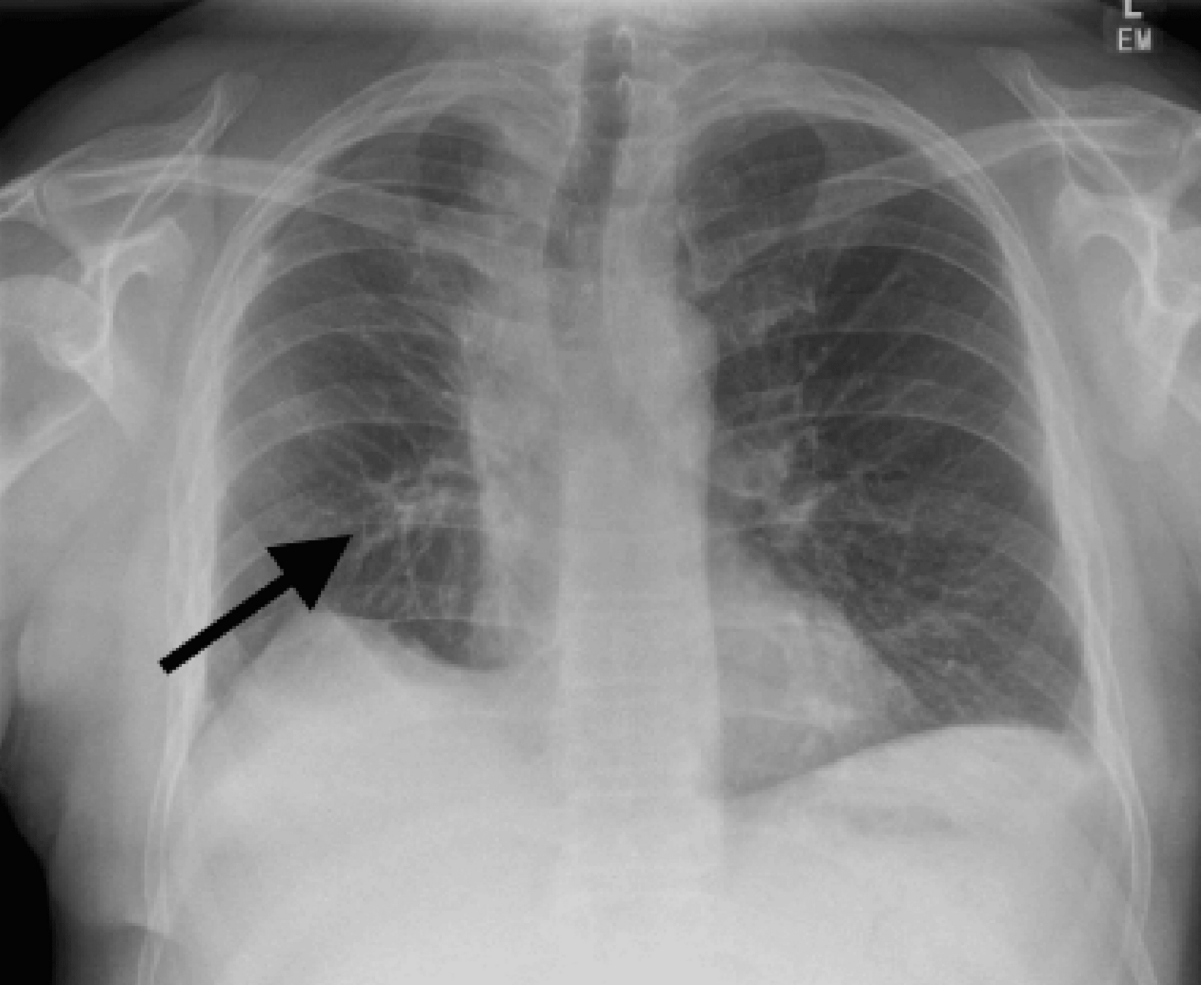
Chest X-ray in PA view, erect, post-operative, demonstrating volume loss in the right hemithorax (black arrow). PA: posteroanterior view.

A 47-year-old man presented after a fall, reporting lower back pain and mild shortness of breath. A chest radiograph (Figure 1) revealed a round density in the right lung hilum, prompting further evaluation. A computerised tomography (CT) scan identified a 6.4-cm right hilar mass and bilateral lung nodules, initially suggestive of a carcinoid tumour (Figure 2). To assess for metastasis, fluorodeoxyglucose positron emission tomography (FDG PET)-CT was performed, showing low-grade uptake and mild somatostatin receptor (SSTR) expression in the mass, with no systemic symptoms (Figure 3). These findings reduced suspicion for atypical carcinoid and raised concern for low-grade lymphoma.

**Figure 4:**
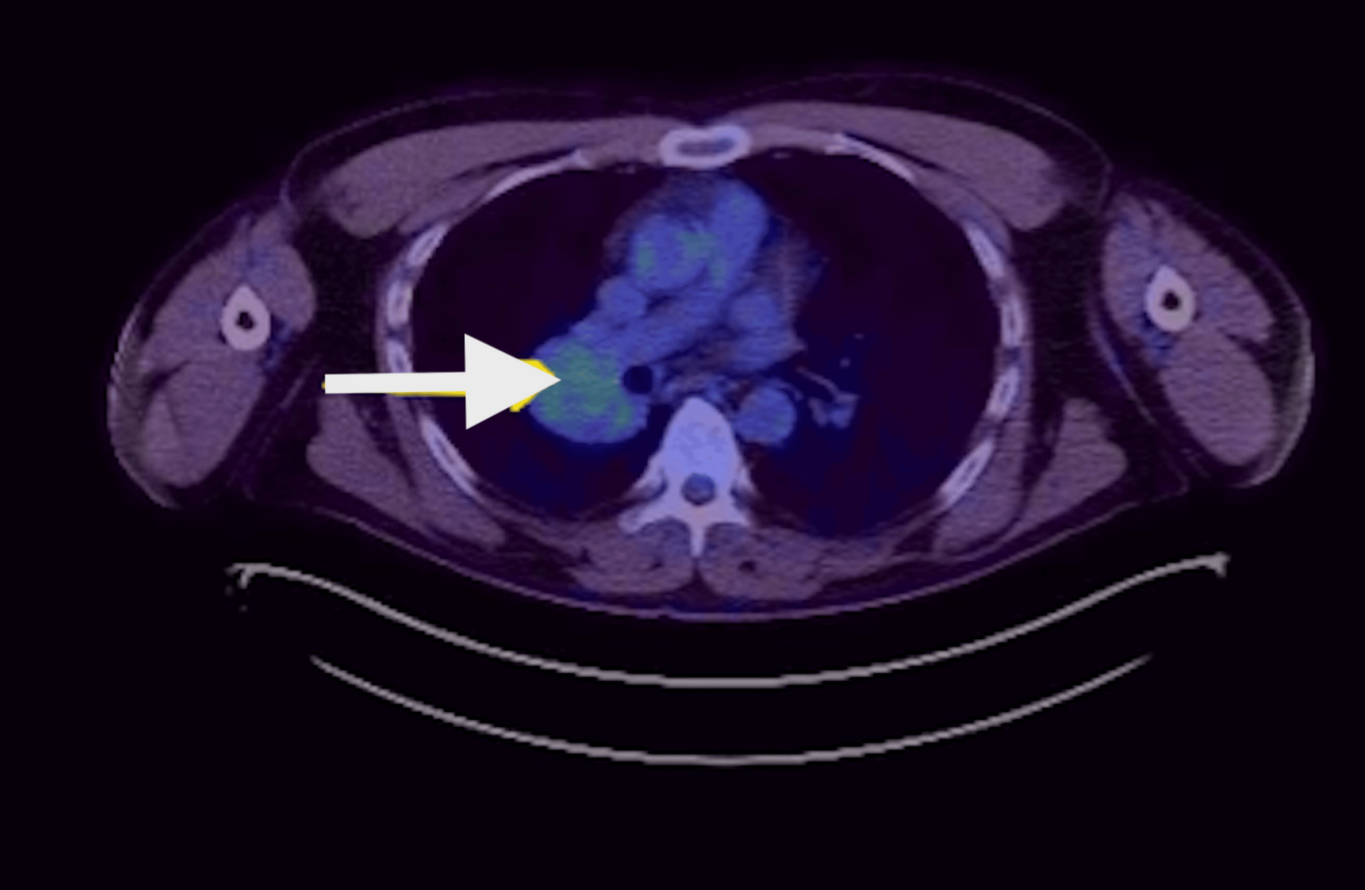
FDG PET-CT in axial view demonstrating low-grade uptake compared to background activity (white arrow). FDG PET: fluorodeoxyglucose positron emission tomography.

Endobronchial ultrasound (EBUS)-guided fine-needle aspiration cytology (FNAC) of the mass, which was inseparable from an enlarged hilar lymph node, revealed predominantly lymphocytes, occasional eosinophils, and no malignant cells, suggesting possible lymph node sampling. To clarify the diagnosis, additional investigations were pursued, including gallium-68 (Ga-68) DOTA-[Tyr³]-octreotate (DOTATATE) PET-CT, liver MRI, and navigational bronchoscopy under general anaesthesia. The Ga-68 DOTATATE PET-CT showed low-grade SSTR uptake in the hilar mass and no metastatic disease (Figure 4), corroborated by a normal liver MRI. A CT-guided endobronchial biopsy demonstrated foamy alveolar macrophages, chronic bronchial wall inflammation, and crushed lymphoid aggregates positive for CD45 but negative for CKAE1/AE3 and synaptophysin, with no evidence of dysplasia or malignancy. However, the possibility of a non-representative sample persisted. Pulmonary function tests were normal, such as a forced expiratory volume in 1 second (FEV1) of 85%, a forced vital capacity (FVC) of 92%, and a transfer factor for carbon monoxide (TLCO) of 96%.

Despite an extensive workup, the diagnosis remained elusive. The thoracic surgery team recommended bronchoscopy, right thoracotomy, and possible pneumonectomy for definitive diagnosis and treatment, while also discussing alternatives like observation or radiotherapy with the patient. During video-assisted thoracoscopic surgery (VATS), a large hilar mass was found in the upper lobe, adherent to the middle lobe, lower lobe, and bronchus intermedius. A right posterolateral thoracotomy at the fifth intercostal space was performed, and frozen section analysis of station 10 (lower lobe) was negative. An en bloc bilobectomy, including lymph node stations 8, 10, 11, and 4R, was completed.

**Figure 5:**
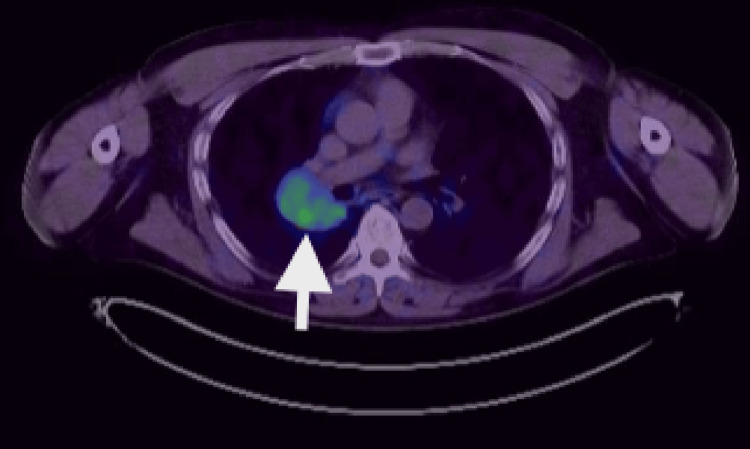
Nuclear medicine Ga-68 DOTATATE PET-CT scan in axial view demonstrating low-grade SSTR uptake (white arrow). Ga-68: gallium-68, PET: positron emission tomography, SSTR: somatostatin receptor.

Histopathological examination confirmed unicentric Castleman disease (UCD). The patient recovered well post-operatively, with normal chest X-ray findings and a stable apical space after chest drain removal and expected volume loss post-operatively (Figure 5). A surveillance plan was established during follow-up visits.

## Discussion

This case report highlights the diagnostic complexities associated with unicentric Castleman disease (UCD), particularly when it presents as a hypervascular mass with inconclusive imaging and biopsy findings. The definitive diagnosis of UCD was only established after a two-lobe lung resection, underscoring the challenges in pre-operative diagnosis and the extent of intervention required.

Diagnostic challenges and imaging findings

As reported in the *American Journal of Hematology* by Dispenzieri and Fajgenbaum [5], Castleman disease (CD) is a rare lymphoproliferative disorder that can mimic both benign and malignant conditions, including lymphomas and neuroendocrine tumours (NETs) (National Organization for Rare Disorders (NORD)-Castleman Disease). In this case, the hypervascular nature of the mass on CT, combined with bilateral lung nodules, initially suggested a pulmonary carcinoid tumour. FDG PET-CT, performed to assess metastasis, showed low-grade uptake, which was inconsistent with the high metabolic activity typically seen in aggressive malignancies or even some cases of CD (FDG PET/CT findings). Similarly, Ga-68 DOTATATE PET-CT, used to evaluate somatostatin receptor (SSTR) expression characteristic of NETs, revealed only mild uptake, further complicating the diagnostic picture. Prior small studies have shown active CD to be avid on an FDG PET-CT and useful for mapping the extent of the disease and monitoring progression, as explained in the *Radiopaedia* article by Weerakkody et al. [9].

The use of both FDG PET and Ga-68 DOTATATE PET in CD evaluation is novel and not standard practice. FDG PET-CT is more commonly employed to assess metabolic activity and distinguish between unicentric and multicentric CD [9]. In one case report by Liu et al. [10] of a pancreatic mass, significant FDG avidity on FDG PET-CT was demonstrated, along with slightly increased SSTR expression on Ga-68 DOTATATE PET. This was initially suggestive of a possible NET, but post-operative histopathological examination of the resected mass was diagnostic for the hypervascular subtype of CD. This contrasts with our study in which there was reduced uptake on both PET-CT and Ga-68 DOTATATE PET, which further supports the premise that histopathological examination is vital, and that a definitive core biopsy or resection is more informative than imaging alone.

Furthermore, Ga-68 DOTATATE PET is typically reserved for NETs due to their high SSTR expression, as outlined in the *Radiopaedia* article by Morgan et al. [11]. This is discussed by Zuo et al. in a case report of a patient with retroperitoneal fibrosis [12]. In that case, there was also avid uptake on both FDG and Ga-68 DOTATATE PET CT scans, which is also in contrast to our case. Reports of its use in CD are scarce, with variable uptake documented, ranging from intense to low, depending on the histological subtype and individual lesion characteristics. The low uptake on both scans in our case is an uncommon combination that broadened the differential diagnosis rather than narrowing it, highlighting the limitations of functional imaging in atypical presentations of CD.

The inability to achieve a pre-operative diagnosis despite multiple biopsies underscores the challenge of diagnosing CD non-invasively. Core needle biopsy or excisional biopsy is often required for definitive diagnosis, as imaging alone is rarely conclusive (imaging of Castleman disease). In retrospect, had CD been considered earlier in the differential diagnosis, additional biopsy attempts or alternative imaging strategies might have been pursued to avoid extensive surgery.

Surgical intervention and clinical implications

The requirement for a two-lobe lung resection to establish the diagnosis is a significant aspect of this case. While surgical excision is the standard treatment for UCD and is typically curative, it is generally considered a last resort due to the benign nature of the condition [5]. The mass’s adherence to multiple lung lobes and the bronchus intermedius necessitated a bilobectomy, which is more extensive than the lobectomy reported in some UCD cases. In a systematic review of intra-pulmonary UCD by Pannu et al. [13], in all 34 recorded cases, attempts at attaining a diagnosis through small-needle biopsies or fresh-frozen samples either failed or were inconclusive, necessitating resection for diagnosis and treatment. Our case highlights the delicate balance between achieving a definitive diagnosis and avoiding overly aggressive interventions for a benign condition.

The successful post-operative outcome, with uneventful recovery and satisfactory follow-up, reinforces the curative potential of complete surgical resection for UCD. However, the extent of surgery prompts discussion about whether a more conservative approach could have been feasible had CD been suspected earlier. For instance, in a comprehensive review of two cases of CD by Swee et al. [14], pre-operative embolization of highly vascular CD tumours had been reported to reduce surgical risks, particularly in mediastinal cases, and could be considered in similar scenarios (imaging and clinical features).

## Conclusions

This case illustrates the diagnostic and therapeutic challenges posed by UCD when it presents with atypical imaging and biopsy findings. The novel use of both FDG PET and Ga-68 DOTATATE PET, while informative, did not resolve the diagnostic uncertainty, leading to a major surgical intervention. By highlighting the complexities of CD diagnosis and the potential for less invasive strategies, this case underscores the need for heightened awareness and consideration of CD in similar clinical scenarios.
